# Early endonuclease-mediated evasion of RNA sensing ensures efficient coronavirus replication

**DOI:** 10.1371/journal.ppat.1006195

**Published:** 2017-02-03

**Authors:** Eveline Kindler, Cristina Gil-Cruz, Julia Spanier, Yize Li, Jochen Wilhelm, Huib H. Rabouw, Roland Züst, Mihyun Hwang, Philip V’kovski, Hanspeter Stalder, Sabrina Marti, Matthias Habjan, Luisa Cervantes-Barragan, Ruth Elliot, Nadja Karl, Christina Gaughan, Frank J. M. van Kuppeveld, Robert H. Silverman, Markus Keller, Burkhard Ludewig, Cornelia C. Bergmann, John Ziebuhr, Susan R. Weiss, Ulrich Kalinke, Volker Thiel

**Affiliations:** 1 Department of Infectious Diseases and Pathobiology, University of Bern, Bern, Switzerland; 2 Federal Department of Home Affairs, Institute of Virology and Immunology, Bern and Mittelhäusern, Switzerland; 3 Institute of Immunobiology, Kantonsspital St.Gallen, St.Gallen, Switzerland; 4 Institute for Experimental Infection Research, TWINCORE, Centre for Experimental and Clinical Infection Research, a joint venture between the Helmholtz Centre for Infection Research and the Hannover Medical School, Hannover, Germany; 5 Department of Microbiology, Perelman School of Medicine, University of Pennsylvania, Philadelphia, PA, United States of America; 6 Universities Giessen & Marburg Lung Center (UGMLC), Deutsches Zentrum für Lungenforschung (DZL), Giessen, Germany; 7 Virology Division, Department of Infectious Diseases and Immunology, Faculty of Veterinary Medicine, Utrecht University, Utrecht, The Netherlands; 8 Singapore Immunology Network, Singapore; 9 Department of Neurosciences, Lerner Research Institute, Cleveland Clinic Foundation, Cleveland, Ohio, United States of America; 10 Graduate School for Biomedical Science, University of Bern, Bern, Switzerland; 11 Max-Planck-Institute of Biochemistry, Martinsried, Germany; 12 Washington University School of Medicine, St. Louis, MO, USA; 13 Institute for Medical Virology, Justus-Liebig-University, Giessen, Germany; 14 Department of Cancer Biology, Lerner Research Institute, Cleveland, Ohio, United States of America; 15 Institute of Novel and Emerging Infectious Diseases, Friedrich-Loeffler-Institut, Greifswald-Insel Riems, Germany; University of Iowa, UNITED STATES

## Abstract

Coronaviruses are of veterinary and medical importance and include highly pathogenic zoonotic viruses, such as SARS-CoV and MERS-CoV. They are known to efficiently evade early innate immune responses, manifesting in almost negligible expression of type-I interferons (IFN-I). This evasion strategy suggests an evolutionary conserved viral function that has evolved to prevent RNA-based sensing of infection in vertebrate hosts. Here we show that the coronavirus endonuclease (EndoU) activity is key to prevent early induction of double-stranded RNA (dsRNA) host cell responses. Replication of EndoU-deficient coronaviruses is greatly attenuated *in vivo* and severely restricted in primary cells even during the early phase of the infection. In macrophages we found immediate induction of IFN-I expression and RNase L-mediated breakdown of ribosomal RNA. Accordingly, EndoU-deficient viruses can retain replication only in cells that are deficient in IFN-I expression or sensing, and in cells lacking both RNase L and PKR. Collectively our results demonstrate that the coronavirus EndoU efficiently prevents simultaneous activation of host cell dsRNA sensors, such as Mda5, OAS and PKR. The localization of the EndoU activity at the site of viral RNA synthesis–within the replicase complex—suggests that coronaviruses have evolved a viral RNA decay pathway to evade early innate and intrinsic antiviral host cell responses.

## Author summary

Coronaviruses are long known to be particularly successful in evading host innate immune responses. This manifests in barely detectable interferon responses during the first hours of infection and greatly facilitates establishment of robust virus replication. This phenotype of early innate immune evasion is common to all coronaviruses and can have detrimental consequences particular for infections with highly pathogenic coronaviruses, such as SARS-CoV and MERS-CoV. We therefore hypothesized that there must be an evolutionary conserved viral function that has evolved to prevent sensing of coronavirus infection by infected host cells. Our study now describes this function, namely the highly conserved coronavirus endoribonuclease activity. We found that coronaviruses that lack this enzymatic activity are readily visible to infected host cells that can now mount a swift and potent host response restricting virus replication within hours. Our study provides a new paradigm of a first layer of RNA virus innate immune evasion during the early phase of infection, that takes place at the site of RNA synthesis, and is based on removal of dsRNA that would otherwise trigger the simultaneous activation of cytoplasmic host cell sensors.

## Introduction

Host innate immune responses are of particular importance during the early phase of virus infection to restrict virus replication and spread. They rely on the ability to differentiate between immunological “self” and “non-self” in order to swiftly activate diverse antiviral effector mechanisms. Conceptually, sensing of virus infection is mainly mediated through recognition of viral nucleic acids, which are considered to comprise pathogen-associated molecular patterns (PAMPs) that are recognized by specialized host cell pathogen recognition receptors (PRRs) [1]. Double-stranded (ds) RNA, an obligate replication intermediate of positive-stranded RNA viruses that is accumulating during replication, is known as an important PAMP within the cytoplasm of infected cells. Host cell responses to dsRNA are versatile and include the expression of IFN-I by activating RIG-I like helicases (RLRs), such as Rig-I and Mda5, the inhibition of host cell translation by activating PKR, and the degradation of viral and host cell-derived RNA by activating the OAS/RNase L pathway [2].

Coronaviruses are positive-stranded RNA viruses that replicate in the host cell cytoplasm. They are well known to evade innate immune activation, particularly during the early phase of the infection [3–6]. Coronavirus innate immune evasion is multifaceted and involves ribose-2’-O methylation of viral RNA, as well as compartmentalised RNA synthesis at virus-induced membrane structures comprised of convoluted membranes and double membrane vesicles [7–9]. The importance of functions encoded by the CoV replicase gene is further exemplified by non-structural protein (nsp) 1 that suppresses host gene expression by mediating host mRNA degradation [10, 11], and nsp3 that contains a papain-like proteinase with deubiquitination activity interfering with IFN-I host cell responses [12, 13]. In addition, a number of accessory gene functions, although less conserved, have been described to target downstream events of innate immune activation, such as a phosphodiesterase (PDE) activity encoded by some coronavirus strains, which degrades 2’,5’-oligoadenylate messenger molecules essential for RNase L activation [14, 15].

Here we addressed a possible role of the highly conserved coronavirus EndoU activity in innate immune evasion. The EndoU domain is harboured in non-structural protein (nsp) 15 that is considered as an integral component of the coronaviral replicase-transcriptase complex (RTC)[16–19]. By using immunofluorescence microscopy analyses in HCoV-229E-infected cells with a HCoV-229E-nsp15-specific monoclonal antibody the characteristic perinuclear staining pattern known from various other CoV nsps was reported [19]. For MHV-A59, a similar study reports MHV-nsp15-specific perinuclear puncta that were detected using an MHV-nsp15-specific rabbit antiserum that partially overlapped with MHV nucleocapsid staining in MHV-A59-infected cells [20, 21]. Moreover, MHV nsp15 was shown to co-localize with viral RNA and to fractionate in similar fractions as other nsps following MHV infection [21, 22]. Notably, upon ectopic expression of a fusion protein comprised of the green fluorescent protein (GFP) and MHV-nsp15, a pattern of cytoplasmic speckles, distinct from the characteristic pattern of the CoV replicase complex was observed, suggesting that the localization of ectopically expressed nsp15 or GFP-nsp15 fusion proteins may differ from the localization of nsp15 that is expressed in the context of the CoV polyprotein 1ab [20]. The CoV EndoU has uridylate-specific endonucleolytic activity on single-stranded and dsRNA[17] and is related to (i) cellular enzymes prototyped by XendoU[16, 23] and (ii) viral homologs conserved in all nidoviruses known to infect vertebrate hosts including fish, birds and mammals, suggesting an important role for this enzyme in an ancient cellular pathway. Over the past years, a wealth of structural and biochemical information has been obtained for EndoU. However, the precise role of this virus-encoded nucleolytic activity in coronavirus/nidovirus replication remains enigmatic [17, 18, 24–26]. Surprisingly, although EndoU is coexpressed with other key replicative proteins as part of the viral replicase polyprotein, its enzymatic activity is not essential for viral RNA synthesis in most cell culture systems[26]. In this work we illustrate a pronounced impact of the coronavirus EndoU activity on innate immune evasion. Specifically, we show that genetically engineered mutants of mouse hepatitis virus (MHV) and human coronavirus 229E (HCoV-229E), respectively, that encode an EndoU active-site substitution known to abolish nucleolytic activity, were severely attenuated and elicited an immediate and simultaneous activation of host cell dsRNA sensors.

## Results

### Severe attenuation of EndoU-deficient coronaviruses

**Fig 1:**
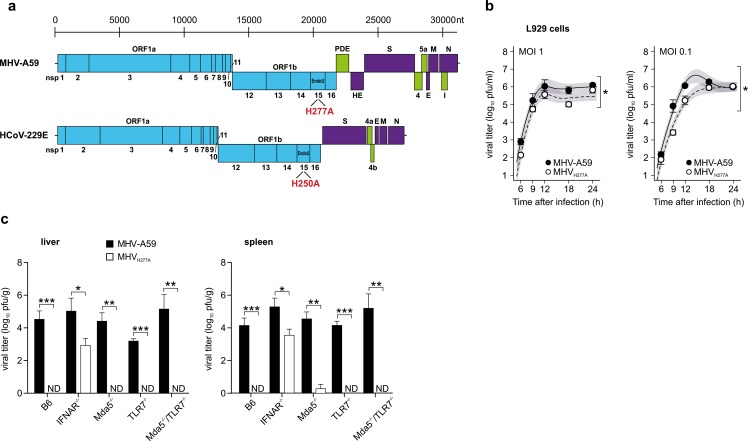
The CoV endoribonuclease is essential for replication and spread in vivo. (**a**) Genome organization of the EndoU-deficient murine hepatitis virus (MHV) with an active site His to Ala substitution (MHV_H277A_) and a corresponding human coronavirus 229E mutant (HCoV-229E_H250A_) in the non-structural protein 15. (**b**) Replication kinetics of MHV-A59 and MHV_H277A_ in murine L929 fibroblasts after infection at a MOI of 1 and 0.1, presented as viral titer in plaque forming units (pfu). Data represent two independent experiments, each performed in duplicates. Mean and SEM are depicted. The 95% confidence band is highlighted in grey. The differences in peak levels of viral titers were calculated by using the non-linear regression model described in Material and Methods (peak MHV-A59: 6.0, MHV_H277A_: 5.6, p = 0.024, left panel; peak MHV-A59: 6.6, MHV_H277A_: 6.0, p = 0.016, right panel) and significance is displayed as * p < 0.05. (**c**) Viral titers of MHV-A59 and MHV_H277A_ in liver and spleen of C57BL/6, IFNAR^-/-^, Mda5^-/-^, TLR7^-/-^, and Mda5^-/-^/TLR7^-/-^ mice at two days post intraperitoneal infection (500 pfu). Data represent three to four independent experiments, each based on two to three mice per strain and virus. Mean and SEM are depicted. Data points that show significant differences in a two-sided, unpaired Student’s t-test are displayed; * p < 0.05, ** < 0.01, *** < 0.001. ND, not detected.

Based on biochemical and structural information on coronavirus EndoU active-site residues[17, 27, 28], we constructed EndoU-deficient mutants of HCoV-229E and MHV (HCoV-229E_H250A_ and MHV_H277A_) and assessed their replication characteristics *in vitro* and *in vivo* (Fig 1A). Replication of MHV_H277A_ was reduced in L929 cells, but peak titers almost reached those of wild-type MHV-A59, confirming that the coronavirus EndoU activity is dispensable for virus replication *in vitro* (Fig 1B)[25, 26]. In sharp contrast, compared to wild-type MHV-A59, the EndoU-deficient MHV_H277A_ was severely attenuated *in vivo* (Fig 1C). MHV_H277A_ replication was not detectable in spleen and liver of C57BL/6 mice at two days post intraperitoneal infection with 500 plaque-forming units (pfu), demonstrating that the EndoU activity is required for efficient replication and spread *in vivo*. Notably, replication and spread of MHV_H277A_ was partly restored in mice deficient for the IFN-I receptor (IFNAR), with viral titers of MHV_H277A_ in the spleen and liver of IFNAR-deficient mice that did not reach those of MHV-A59. Interestingly, concerning the role of Mda5 and TLR7, which are known as main cytoplasmic and endosomal PRRs for coronaviral RNA, respectively, MHV_H277A_ replication was not restored in Mda5-deficient, TLR7-deficient, or Mda5- and TLR7-deficient mice. This phenotype clearly differs from that described for coronaviruses that lack ribose-2’-O methyl-transferase (OMT) activity [9]. Thus, in experiments reported previously, we found that replication of OMT-deficient MHV (MHV_D130A_) is restored in mice that are deficient for Mda5 and TLR7, suggesting that lack of ribose-2’-O methylation is tolerated if these two RNA sensors are absent. The lack of any detectable replication of the EndoU-deficient MHV_H277A_ in Mda5- and TLR7-deficient mice therefore indicates that Mda5- and TLR7-mediated IFN-I expression may not exclusively restrict MHV_H277A_ replication and that other mechanisms contribute to the observed attenuation of MHV_H277A_ replication.

### EndoU-deficiency results in increased IFN-β expression

**Fig 2:**
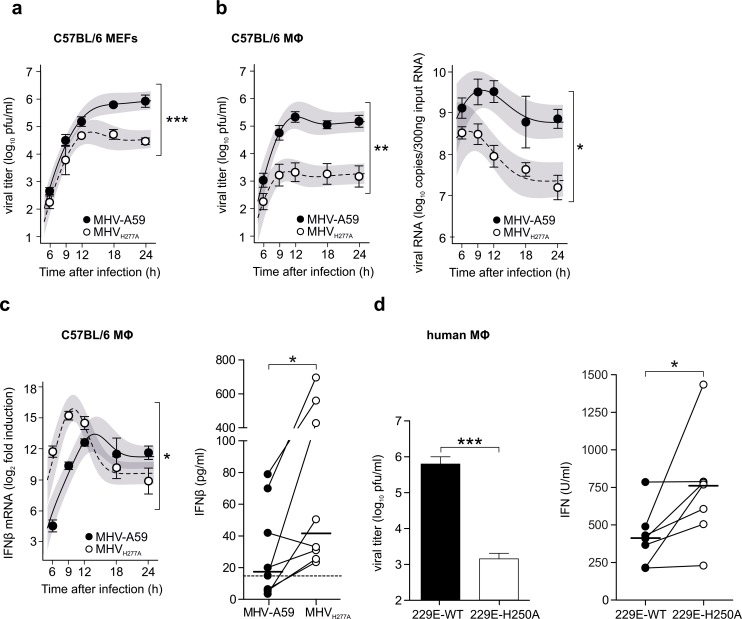
EndoU-deficient coronaviruses are severely attenuated in primary macrophages and trigger an elevated IFN-I response. (**a**) Replication kinetics of MHV-A59 and MHV_H277A_ in C57BL/6 mouse embryonic fibroblasts (MEFs) after infection at a MOI of 1, presented as viral titer in pfu. Data represent four independent experiments, each performed in two to four replicas. The difference in peak levels of viral titers (peak MHV-A59: 5.9, peak MHV_H277A_: 4.8) was statistically significant (***, p<0.001). (**b**) Replication kinetics of MHV-A59 and MHV_H277A_ (left panel; titers in pfu) and cell-associated viral RNA (right panel; qRT-PCR) following infection of C57BL/6 bone marrow-derived macrophages (MOI = 1). Data represent eight (left panel) or five (right panel) independent experiments, each performed in two to three replicas. The difference in peak levels of viral titers (left panel: peak MHV-A59: 5.3, MHV_H277A_: 3.3) and the difference in peak levels of RNA copies (right panel: peak MHV-A59: 9.5, MHV_H277A_: 8.5) were statistically significant (p = 0.002, p = 0.018, respectively). (**c**) Expression of IFN-β mRNA (left panel; qRT-PCR) and protein (right panel; ELISA) in C57BL/6 macrophages following infection of MHV-A59 and MHV_H277A_ (MOI = 1). Expression of IFN-β mRNA was normalized to levels of the household genes GAPDH and Tbp and is displayed relative to mock as ΔΔC_T_. The IFN-β ELISA detection limit is indicated with a dashed line. Data represent seven (left panel) or eight (right panel) independent experiments, each performed in two to three replicas. The difference in peak levels of IFN-β mRNA (MHV-A59: 13.4, MHV_H277A_: 15.8) was statistically significant (p = 0.04). Significance of IFN-β protein at 9 h.p.i. was assessed by a two-sided, Wilcoxon matched-pairs test (p = 0.016). **(a-c)** Mean and SEM are depicted. The 95% confidence band is highlighted in grey. Statistically significant comparisons are displayed; * p < 0.05, ** < 0.01, *** < 0.001. (**d**) Titers (pfu) of HCoV-229E wild type and HCoV-229E_H250A_ (left panel) and expression of IFN-I (right panel; IFN-I bioassay) in human blood-derived macrophages, 24 hours after infection (MOI = 1). Data represent six (left panel) or seven (right panel) independent experiments, each performed in three to four replicas. Significance was assessed by a two-sided, unpaired Student’s t-test (left panel, p<0.001) and a Wilcoxon matched-pairs test (right panel; p = 0.016). **(c-d)** Mean and SEM are depicted. Statistically significant comparisons are displayed; * p < 0.05, *** < 0.001.

The severe attenuation of MHV_H277A_ growth *in vivo* prompted us to assess MHV_H277A_ and HCoV-229E_H250A_ replication in primary target cells. As shown in Fig 2A, MHV_H277A_ replication in primary murine embryonic fibroblasts (MEFs) was comparable to that of MHV-A59 until 9–12 hours post infection (h.p.i.), but was significantly restricted later during the infection. Moreover, replication of MHV_H277A_ was even more severely reduced in bone marrow-derived murine macrophages, and accompanied by early induction of IFN-β expression (Fig 2B and 2C). Notably, levels of IFN-β mRNA were only transiently (6 to 12 h.p.i.) elevated in MHV_H277A_ compared to MHV-A59 infected macrophages, and declined along with viral titers and viral RNA during the late phase of infection. Likewise, replication of the EndoU-deficient HCoV-229E_H250A_ was severely restricted in human blood-derived macrophages (Fig 2D). We observed significantly elevated IFN-I expression in a panel of human macrophages derived from seven individual donors after infection with HCoV-229E_H250A_ compared to wild-type HCoV-229E infection (Fig 2D), consistent with reduced viral replication.

**Fig 3:**
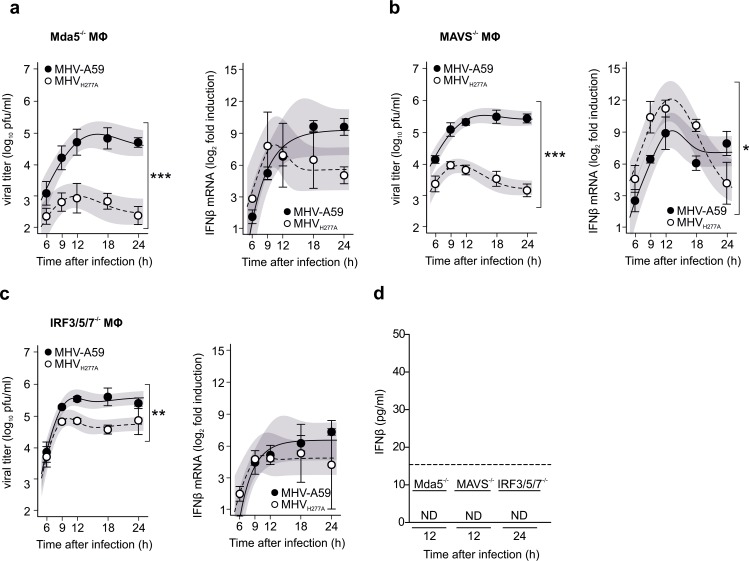
Replication of MHV_H277A_ in primary macrophages with deficiencies in the IFN-I induction pathway. Kinetics of viral replication (left panels) and IFN-β mRNA (right panels) in bone marrow-derived macrophages deficient for Mda5 **(a)**, MAVS **(b)** and in macrophages triple knockout for IRF3, IRF5 and IRF7 **(c)** following infection with MHV-A59 and MHV_H277A_ (MOI = 1). (**d**) IFN-β in the supernatant of infected macrophages was measured using an ELISA. All values were outside of the detection limit of 15.6 pg/ml (dashed line) and thus depicted as ND (not detected). (**a-d**) Data represent three independent experiments, each performed in two to three replicas. **(a)** The difference in peak levels of viral titers (MHV-A59: 4.9, MHV_H277A_: 3.0) was statistically significant (p<0.001), the difference in peak levels of IFN-β expression (MHV-A59: 9.2, MHV_H277A_: 7.8) was statistically not significant (p = 0.44). **(b)** The differences in peak levels of viral titers (MHV-A59: 5.5, MHV_H277A_: 3.9) and IFN-β expression (MHV-A59: 9.1, MHV_H277A_: 12.1) were statistically significant (p<0.001, p = 0.024, respectively). **(c)** The difference in peak levels of viral titers (MHV-A59: 5.5, MHV_H277A_: 4.9) was statistically significant (p = 0.002). The difference in peak levels of IFN-β expression (MHV-A59: 6.5, MHV_H277A_: 5.1) was statistically not significant (p = 0.368). Mean and SEM are depicted. The 95% confidence band is highlighted in grey. Statistically significant comparisons are displayed; * p < 0.05, ** < 0.01, *** < 0.001.

Next, we addressed if, and to what extent, the growth defects observed for EndoU-deficient coronaviruses correlate with the induction of IFN-β expression. Mda5 has been described as the main RNA sensor of coronavirus infection in murine macrophages [29]. Compared to wild-type macrophages, IFN-β expression was reduced in Mda5-deficient macrophages following MHV_H277A_ infection, as shown by qRT-PCR and IFN-β ELISA (Fig 3A and 3D). Surprisingly, and again in contrast to the phenotype of the OMT-deficient MHV_D130A_ [9], MHV_H277A_ replication was not restored in Mda5-deficient macrophages (Fig 3A). Similarly, although IFN-β expression was likewise reduced in MAVS-deficient macrophages, MHV_H277A_ replication was not restored in MAVS-deficient macrophages (Fig 3B and 3D). Even in IRF3/IRF5/IRF7 (IRF3/5/7 ^-/-^) triple-knockout macrophages, that display an almost negligible induction of IFN-β expression, MHV_H277A_ replication was not fully restored (Fig 3C and 3D). IFN-β protein assessed by IFN-β ELISA was below detection in all three macrophage genotypes (Mda5^-/-^, MAVS^-/-^, IRF3/5/7^-/-^; Fig 3D). These results indicate that MHV_H277A_ replication is either highly sensitive to already marginal amounts of IFN-I, or that other antiviral host cell responses may contribute to the attenuation of MHV_H277A_.

### EndoU-deficient coronaviruses display a pronounced sensitivity to IFN-I treatment

**Fig 4:**
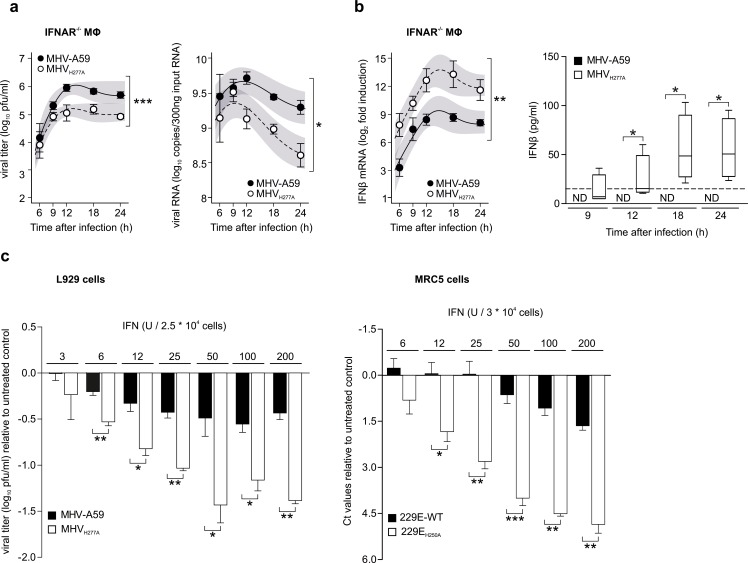
Replication of EndoU-deficient MHV is partially restored in IFNAR^-/-^ macrophages and EndoU mutants display a pronounced sensitivity to IFN-I treatment. (**a**) Replication kinetics of MHV-A59 and MHV_H277A_ (left panel; titers in pfu) and cell-associated viral RNA (right panel; qRT-PCR) following infection of IFNAR^-/-^ bone marrow-derived macrophages (MOI = 1). Data represent four independent experiments, each performed in two to three replicas. Mean and SEM are depicted. The 95% confidence band is highlighted in grey. The differences in peak levels of viral titers (MHV-A59: 6.0, MHV_H277A_: 5.2) and RNA copies (MHV-A59: 9.7, MHV_H277A_: 9.3) were statistically significant (p<0.001, p = 0.032, respectively). (**b**) Expression of IFN-β mRNA (left panel; qRT-PCR) and protein (right panel; ELISA) in IFNAR^-/-^ macrophages following infection of MHV-A59 and MHV_H277A_ (MOI = 1). Data represent four (left panel) and three (right panel) independent experiments, each performed in two to three replicas. Median and the 1–99 percentiles are displayed. Dashed line depicts limit of detection (right panel). The difference in peak levels of IFN-β expression (MHV-A59: 9.4, MHV_H277A_: 13.8) was statistically significant (p = 0.002). Significance of IFN-β expression was assesses by a Wilcoxon matched-pairs test, * p < 0.05. ND, not detected. (**c**) Sensitivity of wild type and EndoU-deficient MHV (left panel) and HCoV-229E (right panel) viruses to IFN-I pre-treatment (4 h) in L929 cells (left panel) and MRC-5 cells (right panel) with various dosages of IFN-I (MOI = 1). Virus replication was measured at 24 h.p.i. by plaque assay (MHV) and at 48 h.p.i. by qRT-PCR (HCoV-229E), respectively. Data represent three independent experiments, each performed in two to three replicas. Data are displayed as differences to untreated controls and statistical comparisons between wild type and EndoU-deficient viruses were performed for each concentration. Mean and SEM are displayed. Data points that show significant differences in a two-sided, unpaired Student’s t-test are depicted. * p < 0.05, ** p < 0.01 and *** p < 0001.

In order to address the sensitivity of EndoU-deficient coronaviruses to IFN-I, we first assessed MHV_H277A_ replication in IFNAR-deficient macrophages. As shown in Fig 4A, MHV_H277A_ replication was partially restored to levels that almost reached those of wild-type MHV-A59 replication. Importantly, IFN-β expression was elevated in IFNAR-deficient macrophages that had been infected with MHV_H277A_ compared to those infected with wild-type MHV-A59, demonstrating that increased expression of IFN-β can be uncoupled from attenuation of MHV_H277A_ (Fig 4B). We also noted that IFN-β expression was delayed in IFNAR-deficient macrophages following MHV_H277A_ infection compared to wild-type macrophages. This observation is in agreement with previous reports that suggested a macrophage-specific autocrine IFN-β priming loop in wild-type macrophages enhances cytokine and chemokine expression following MHV infection [30].

The severe attenuation of MHV_H277A_ and HCoV-229E_H250A_ replication in wild-type murine and human macrophages, respectively, precluded the use of primary macrophages to assess the sensitivity of EndoU-deficient coronaviruses to IFN-I pre-treatment. Therefore, we infected murine L929 cells and human MRC5 lung fibroblasts with MHV_H277A_ and HCoV-229E_H250A_, respectively, and applied different dosages of IFN-I for 4 hours prior to infection. Compared to wild type MHV and HCoV-229E, respectively, both EndoU-deficient viruses indeed displayed a pronounced sensitivity to IFN-I pre-treatment (Fig 4C). Remarkably, MHV_H277A_ displayed a sensitivity to IFN-I treatment that is comparable to that of the highly IFN-I sensitive OMT-deficient mutant MHV_D130A_ (S1 Fig). However, compared to the OMT-deficient mutant MHV_D130A_ the phenotype of the EndoU-deficient MHV_H277A_ differs mainly in the lack of restoration of replication under conditions with strongly reduced IFN-I expression (e.g. in Mda5^-/-^ macrophages; Fig 3A), suggesting that other, most likely IFN-I inducible, antiviral effector mechanisms account for restriction of MHV_H277A_ replication. Collectively, these results demonstrate that the coronavirus EndoU activity plays a pivotal role in innate immune evasion in the context of the IFN-I system.

### EndoU-deficient coronaviruses induce activation of the OAS/RNase L pathway

**Fig 5:**
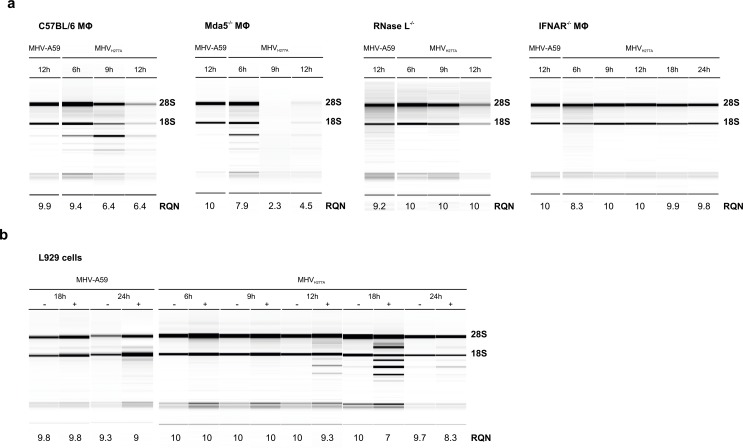
EndoU-deficient MHV induces activation of the OAS-RNase L pathway, resulting in early breakdown of ribosomal RNA. (**a**) Analysis of rRNA integrity in bone marrow-derived macrophages derived from wild type C57BL/6, Mda5^-/-^, RNase L^-/-^, and IFNAR^-/-^ mice following infection with MHV-A59 and MHV_H277A_ (MOI = 1). Total RNA was isolated at indicated time points and degradation of ribosomal RNA as marker for RNase L activation was assessed with a Fragment Analyzer. One representative picture and migration of 18S and 28S ribosomal RNA is displayed. The RNA Quality Number (RQN) is indicated. (**b**) The integrity of rRNA from MHV-A59 and MHV_H277A_ infected (MOI = 1) L929 cells, with or without IFN-I pre-treatment (12.5 U of IFN-I 16h prior to infection). Analysis was performed as in panel (**a**) and one representative image out of five is displayed.

As noted above, coronavirus EndoU-deficiency results in a pronounced sensitivity to IFN-I treatment that is comparable to that of the highly IFN-I sensitive OMT-deficient mutant MHV_D130A_. However, replication of MHV_H277A_ was not restored in Mda5-deficient macrophages. This observation prompted us to consider that replication of EndoU-deficient coronaviruses may activate additional dsRNA-triggered antiviral pathways. We therefore assessed the integrity of ribosomal RNA (rRNA), a marker for the activation of the OAS/RNase L pathway [31], during MHV_H277A_ infection in primary murine macrophages. Indeed, the breakdown of rRNA in MHV_H277A_ infected wild-type macrophages was readily detectable as early as 6–12 h.p.i., thus coinciding with the induction of IFN-β expression during the early phase of the infection (compare Figs 5A and 2C). This finding is highly surprising since MHV-A59 encodes a PDE activity that has been shown to degrade 2’,5’-oligoadenylate messenger molecules essential for RNase L activation [14]. However, the PDE activity was apparently not sufficient to prevent RNase L activation in macrophages that had been infected with EndoU-deficient MHV_H277A_. To exclude that the lack of EndoU activity may directly impact on viral RNA synthesis and lead to reduced levels of subgenomic mRNAs, we assessed the level of genomic and subgenomic mRNA2 (encoding the PDE activity) by qRT-PCR. As shown in S2 Fig, genomic RNA and subgenomic mRNA2 were equally reduced in MHV_H277A_ infected wild-type macrophages, suggesting that the lack of EndoU activity does not result in selective reduction of subgenomic mRNAs. Importantly, while breakdown of rRNA was also readily detectable in Mda5- and MAVS-deficient macrophages, rRNA remained intact in RNase L-deficient macrophages, demonstrating that infection of EndoU-deficient MHV_H277A_ indeed results in the activation of the OAS-RNase L pathway and subsequent degradation of rRNA (Fig 5A; S2 Fig).

Notably, a breakdown of rRNA was not detected in MHV_H277A_–infected IFNAR-deficient macrophages (Fig 5A) concurring with partial restoration of MHV_H277A_ replication in these cells. Accordingly, and as previously published [32, 33], the degree of RNase L activation correlates with levels of OAS expression and we noted indeed reduced baseline expression of OAS 1a, 2 and 3 in IFNAR-deficient compared to wild-type C57BL/6 macrophages (S3 Fig). Likewise, we did not observe breakdown of rRNA in L929 cells (Fig 5B). We therefore assessed the levels of OAS 1a, 2, and 3 expression in L929 with or without IFN-I treatment (12.5 U). As expected, expression of IFN-β was elevated in MHV_H277A_–, but not in MHV-A59-infected L929 cells, irrespectively of IFN-I pre-treatment (S4 Fig). Importantly however, expression of OAS 1a, 2 and 3 in L929 cells was significantly elevated following IFN-I treatment (S4 Fig), and accordingly, rRNA breakdown was readily detectable in IFN-I treated L929 cells that had been infected with MHV_H277A_ (Fig 5B). This data provide evidence for a functional link between the observed pronounced IFN-I sensitivity of MHV_H277A_ and restriction of MHV_H277A_ replication by the OAS/RNase L pathway.

### Involvement of PKR during early replication

**Fig 6:**
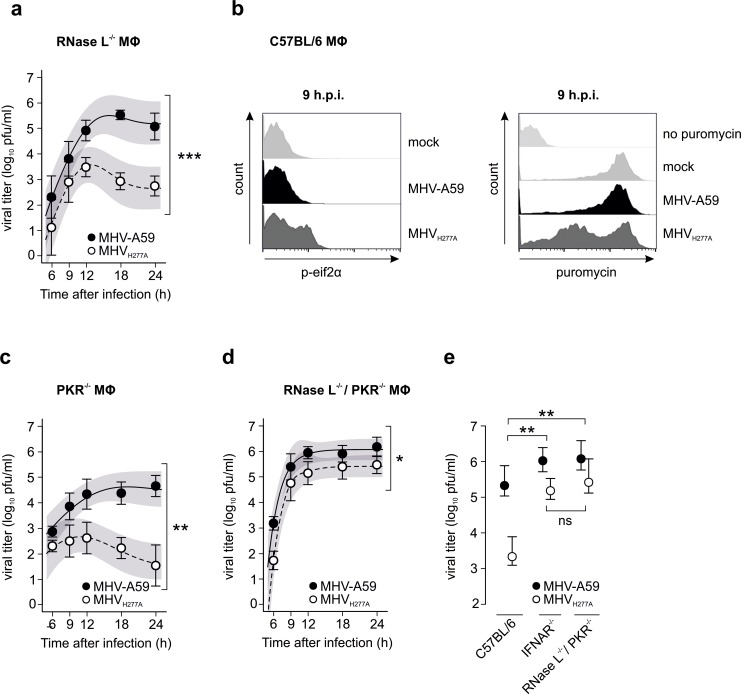
Involvement of RNase L and PKR in restricting replication of EndoU-deficient MHV. **(a)** Replication kinetics of MHV-A59 and MHV_H277A_ following infection (MOI = 1) of bone marrow-derived RNase L^-/-^ macrophages. Mean and SEM are shown. The 95% confidence bands are highlighted in grey. Data represent four independent experiments, each performed in two to three replicas. The difference in peak levels of viral titers (MHV-A59: 5.5, MHV_H277A_: 3.5) was statistically significant (***, p<0.001). **(b)** Intracellular staining of p-eif2α (left panel) and puromycin (right panel) and FACS analysis of MHV-A59 and MHV_H277A_ infected (MOI = 1) C57BL/6 macrophages. One representative histogram out of three is shown. Phosphorylation of eif2α was determined using an antibody directed against p-eif2α. Cells without virus infection (mock) were used as controls (left panel). To label active translation (right panel), puromycin was added to the cells 15min prior to harvesting. Cells without puromycin-treatment (no puromycin) as well as cells without virus infection (mock) were used as controls. **(c, d)** Replication kinetics of MHV-A59 and MHV_H277A_ following infection (MOI = 1) of bone marrow-derived PKR^-/-^ **(c)** and RNase L^-/-^/PKR^-/-^ **(d)** macrophages. Mean and SEM are shown. The 95% confidence bands are highlighted in grey. Data in **(c)** represent three independent experiments, each performed in two to three replicas. The difference in peak levels of viral titers (MHV-A59: 4.6, MHV_H277A_: 2.7) was statistically significant (**, p = 0.004). Data in **(d)** represent three independent experiments, each performed in two to three replicas. The difference in peak levels of viral titers (MHV-A59: 6.1, MHV_H277A_: 5.4) was statistically significant (*, p = 0.036). **(e)** Comparison of differences in peak titers calculated by using the non-linear regression model. Mean and 95% confidence intervals of calculated peak titers of MHV-A59 and MHV_H277A_ following infection (MOI = 1) of bone marrow-derived C57BL/6 (data correspond to Fig 2B), IFNAR^-/-^ (data correspond to Fig 4A) and RNase L^-/-^/PKR^-/-^ (data correspond to Fig 6C) macrophages are displayed. Statistical analysis was performed to compare differences of calculated MHV-A59 and MHV_H277A_ peak titers between C57BL/6 and IFNAR^-/-^ (**, p = 0.008), between C57BL/6 and RNase L^-/-^/PKR^-/-^ (**, p = 0.004) and between IFNAR^-/-^ and RNase L^-/-^/PKR^-/-^ (p = 0.612; ns) macrophages following MHV-A59 and MHV_H277A_ infection.

Surprisingly however, MHV_H277A_ replication was not restored in RNase L-deficient macrophages despite the fact that rRNA remained intact during the entire replication cycle (compare Fig 5A and Fig 6A). This strongly suggests that yet another antiviral pathway, in addition to OAS/RNase L, is activated during MHV_H277A_ infection. One obvious candidate is PKR, a kinase that can be directly activated by dsRNA to phosphorylate the eukaryotic initiation factor 2α (eIF2α), resulting in translation inhibition of cellular and viral mRNAs. Indeed, as shown in Fig 6B (left panel), we readily detected phosphorylated eIF2α at 9 h.p.i.. In addition, we assessed the extent of translational inhibition at 9 h.p.i. by using puromycin and subsequent FACS analysis. As shown in Fig 6B, wild-type MHV-A59 infected cells showed active translation comparable to mock infected macrophages, while translational inhibition was observed in MHV_H277A_–infected macrophages. Finally, we assessed replication of MHV-A59 and MHV_H277A_ in PKR-deficient macrophages, and as shown in Fig 6C, PKR-deficiency alone was also not sufficient for the restoration of MHV_H277A_ replication. Importantly, however, we observed elevated replication of MHV_H277A_ in primary macrophages that are deficient for both, PKR and RNase L that almost reached that of MHV-A59 (Fig 6D) [34]. In order to more precisely analyse the degree of restoration of MHV_H277A_ replication in IFNAR- and in RNase L/PKR-deficient macrophages, we performed a statistical analysis and compared the differences of calculated MHV-A59 and MHV_H277A_ peak titers between C57BL/6 and IFNAR^-/-^ (p = 0.008), between C57BL/6 and RNase L^-/-^/PKR^-/-^ (p = 0.004) and between IFNAR^-/-^ and RNase L^-/-^/PKR^-/-^ (p = 0.612) (Fig 6E). This result shows that MHV_H277A_ replication is restored to a comparable degree in IFNAR^-/-^ and RNase L^-/-^/PKR^-/-^ macrophages.

Collectively, these results suggest that MHV_H277A_ replication results in the early and simultaneous activation of at least three dsRNA-triggered pathways, namely IFN-β expression via Mda5, and antiviral effectors PKR and OAS/RNase L.

### Infection with EndoU-deficient MHV results in increased cytosolic dsRNA

**Fig 7:**
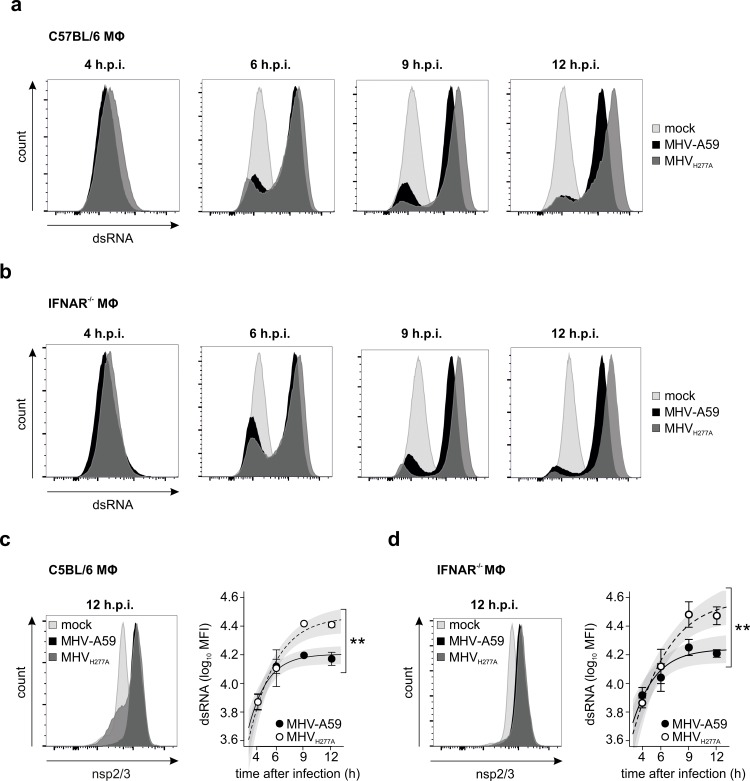
Infection with EndoU-deficient MHV results in increased cytosolic dsRNA. **(a-b)** Intracellular staining of dsRNA and FACS analysis of MHV-A59 and MHV_H277A_ infected (MOI = 1) C57BL/6 **(a)** and IFNAR^-/-^ **(b)** macrophages at 4, 6, 9 and 12 h.p.i.. One representative histogram out of two (a) and three (b) is shown for each time point. Cells without virus infection (mock) were used as controls. **(c-d)** The left panels show cells that were co-stained for MHV-nsp2/3 to control for MHV-A59 and MHV_H277A_ infection. The right panels display the median fluorescent intensity (MFI) of dsRNA peaks detected in **(a-b).** The left panels show data from two (c) and three (d) independent experiments. Cells without virus infection (mock) were used as controls. Mean and SEM are depicted. The 95% confidence band is highlighted in grey. Statistically significant comparisons are displayed (**, p< 0.01).

Since activation of Mda5, PKR and OAS/RNase L are triggered by dsRNA, we assessed if MHV_H277A_ infection results in increased appearance of cytosolic dsRNA. By using the dsRNA-specific antibody J2 for intracellular staining and FACS analysis we assessed the level of dsRNA in MHV-A59- and MHV_H277A_-infected wild-type and IFNAR-deficient macrophages at 4, 6, 9, and 12 h.p.i.. At 4 h.p.i. dsRNA was not yet convincingly detectable in both MHV-A59- and MHV_H277A_-infected wild-type macrophages (Fig 7A). At 6 h.p.i. dsRNA peaks are clearly separated over mock and we observed a slightly stronger dsRNA signal in MHV_H277A_- than in MHV-A59-infected cells. This difference became convincingly apparent and statistically significant at 9 and 12 h.p.i. with dsRNA peaks of MHV_H277A_-infected cells that clearly separated from dsRNA peaks of MHV-A59-infected cells (Fig 7A and 7C, right panel). Importantly, we also controlled for virus infection by staining for the replicase complex (nsp2/3), and as shown in Fig 7C (left panel) the peaks for nsp2/3 from in MHV_H277A_-infected wild-type macrophages did not exceed those of MHV-A59-infected cells. We obtained essentially the same result when we assessed dsRNA and nsp2/3 by FACS analysis following infection of IFNAR^-/-^ macrophages (Fig 7B and 7D), suggesting that dsRNA is also increased in MHV_H277A_-infection under conditions of reduced host cell responses. Collectively, these results demonstrate that cytosolic dsRNA is increased in EndoU-mutant virus infection and suggest that elevated dsRNA is the trigger for the activation of Mda5, PKR, and OAS.

## Discussion

Coronaviruses have long been known to efficiently evade host innate immune responses during the early phase of the infection. However, a defined viral function accounting for the apparent lack of efficient sensing of coronavirus infection has remained elusive. Here we show that the highly conserved coronavirus EndoU activity within the viral RTC plays a major role in providing a first line of innate immune evasion during the early phase of coronavirus infection.

**Fig 8:**
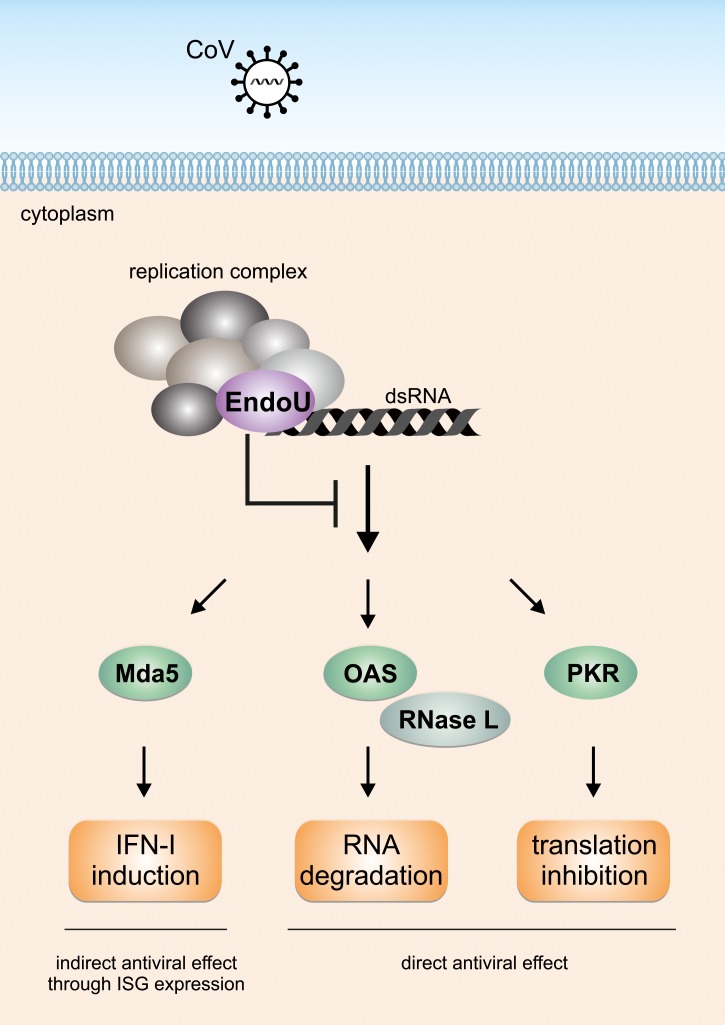
Coronavirus EndoU-mediated innate immune evasion. Following coronavirus infection, the EndoU activity residing in the coronavirus replication complex prevents simultaneous activation of dsRNA sensors Mda5, OAS, and PKR. This strategy allows coronaviruses to efficiently evade antiviral innate host responses such as induction of IFN-I expression, RNase L-mediated RNA degradation, and inhibition of host cell translation.

We show that at least three dsRNA-triggered antiviral pathways are involved in restricting replication of EndoU-deficient coronaviruses (Fig 8). First, infection with EndoU-deficient MHV and HCoV-229E results in rapid Mda5-mediated induction of IFN-β expression. Second, we observe breakdown of ribosomal RNA indicative of activation of the OAS/RNase L pathway that temporally coincides with IFN-β expression. Third, we show that efficient restriction of EndoU-deficient coronaviruses is furthermore dependent on PKR since restoration of EndoU-deficient MHV_H277A_ replication required the absence of both, PKR and RNase L. Our data suggest that direct restriction of replication of EndoU-deficient coronaviruses is mediated by RNase L-mediated RNA degradation and inhibition of host cell translation through activation of PKR. In contrast, the effect of IFN-I appears to be indirect through the induction of ISG expression, that includes OAS/RNase L and PKR. Whether other ISGs may contribute to the restriction of EndoU-deficient coronavirus replication remains to be determined. Finally, we show that MHV_H277A_ replication is associated with increased dsRNA levels during the early phase of the infection, providing a likely PAMP for the observed simultaneous activation of multiple cytoplasmic dsRNA-sensors in cells infected with EndoU-deficient coronaviruses.

The concerted activation of multiple cytoplasmic antiviral pathways strongly suggests sensing of the same PAMP during replication of EndoU-deficient coronaviruses. All three types of sensors, MDA5, OAS1-3, and PKR, are known to recognize dsRNA, suggesting that the PAMP(s) relevant for their activation during infection is/are of viral origin. Notably, RNase L activation can be triggered by different OAS proteins and may depend on particular cell types and virus infections[35]. For example overexpression of OAS 3 was shown to provide RNase L-dependent activity against dengue virus and chikungunya virus infection [36, 37], while OAS 1 and OAS 3 have been implicated in antiviral activity against hepatitis C virus [38]. It recently has been shown that among the human OAS proteins 1, 2, and 3, OAS 3 seems to be mainly responsible for mediating RNase L activation following either polyI:C transfection or virus infection, suggesting a superior role of human OAS 3 over OAS 1 and OAS 2 in restricting virus replication [33]. Interestingly, structural and biochemical studies revealed that OAS 3 is selective for binding of long dsRNA (>50 bp) by involvement of an RNA-binding, but non-catalytic domain, and that OAS 3 is weakly or not activated by short dsRNA or single-stranded RNA, respectively [39]. Likewise, PKR preferentially dimerizes upon binding to dsRNA of similar length (>60 bp) [40, 41] and Mda5 is actually most efficiently activated by even longer dsRNA (>2 kbp) [42] and higher-order structured RNA containing single-stranded and dsRNA [43]. It is thus tempting to propose that viral dsRNA represents the natural substrate of the coronavirus EndoU. However, it remains to be determined which kind of viral dsRNA is cleaved by the EndoU or triggers Mda5, OAS, and PKR activation. Compared to MHV wild-type infection we observed a slight but reproducible increase of dsRNA in MHV_H277A_-infected macrophages by FACS analysis during the early phase of the infection (4–6 h.p.i.) that became more prominent at 9–12 h.p.i., suggesting that the majority of dsRNA is not cleaved by EndoU. This likely includes dsRNA being shielded within double-membrane vesicles and replication intermediates actively involved in viral RNA synthesis that are likely protected by the RTC and the nucleocapsid protein. We therefore speculate that coronaviruses may have evolved a viral RNA quality control mechanism to evade dsRNAs sensing, and that EndoU substrates may comprise dsRNA intermediates within stalled RTCs engaged in genome replication or transcription that are no longer active in viral RNA synthesis.

Within the order Nidovirales, the EndoU domain is highly conserved within the families *Coronaviridae* (comprising two subfamilies *Coronavirinae* and *Torovirinae*) and *Arteriviridae* and has been considered a major genetic marker that discriminates nidoviruses from all other RNA viruses [16–18]. However, the recent discovery of insect nidoviruses (family *Mesoniviridae*) [44, 45] and re-analysis of the ronivirus genome (family *Roniviridae*; infecting crustaceans) revealed that these two nidovirus families do not encode an EndoU domain [44]. In the light of our results it is tempting to speculate that the EndoU domain has evolved in vertebrate nidoviruses (*Corona-* and *Arteriviridae*) to counteract vertebrate-specific innate immune sensing of viral RNA in the context of the type-I interferon system, while the absence of the EndoU domain in roni- and mesoniviruses is indicative of fundamentally different mechanisms of RNA virus innate immune sensing and antiviral effector pathways in invertebrates (e.g. crustaceans and insects) [1].

MHV as a natural mouse pathogen has been instrumental to understand the delicate balance between host IFN-I responses to infection and counteracting mechanisms of coronavirus innate immune evasion. While MHV evades innate immune sensing in most target cells, plasmacytoid dendritic cells (pDCs) remain as major IFN-I producer cells during early coronavirus replication to ensure protection of MHV target cells and control of potentially lethal coronavirus infections [46, 47]. While pDCs sense coronaviral RNA within endosomes through TLR7, our data demonstrate that the coronaviral EndoU delays Mda5-mediated cytoplasmic sensing in macrophages and likely other cell types. This enables coronaviruses to establish robust replication and spread at the entry port of infection. However, in the case of highly pathogenic strains or newly emerging zoonotic coronaviruses, delayed IFN-I responses can have detrimental consequences as recently demonstrated in a murine model of SARS-CoV infection [3]. Early and rapid SARS-CoV replication in the respiratory tract combined with a delayed IFN-I response can result in dysregulated innate immune responses and inflammatory cytokine-driven extensive lung damage. Therefore, antiviral intervention aiming at inhibiting the coronavirus EndoU activity may be a promising approach to restore efficient sensing of coronaviral RNA and thereby activating IFN-I expression as well as antiviral effector pathways such as PKR and OAS/RNase L.

There is a growing number of virus-encoded ribonucleases that have been reported to execute diverse steps in the context of cellular mRNA quality control and mRNA decay [48, 49]. For example, herpesvirus-encoded endonucleases are known to broadly target cellular and also viral mRNAs that are subsequently further processed by cellular exonucleases, such as Xrn1, in order to broadly restrict host cell gene expression [50]. While this strategy indirectly impacts host cell innate immune responses, a more specific interaction of RNA decay and host cell dsRNA responses has recently been described for vaccinia virus decapping enzymes D9 and D10 [51, 52]. They remove 5’-cap structures of partially overlapping vaccinia virus mRNAs that arise during the late phase of infection in order to preclude any accumulation of viral dsRNA and subsequent activation of dsRNA sensors such as PKR and OAS. Notably, also in this case Xrn1 is required to further process the de-capped mRNAs. Our data show that early during coronavirus infection the EndoU activity conceptually fulfils the same task, namely the removal of dsRNA that would otherwise trigger host cell dsRNA responses, such as IFN-β expression and activation of PKR and OAS/RNase L. It will be interesting to address whether Xrn1 is involved in further degrading the coronavirus EndoU cleavage products or if this function could be fulfilled by the coronavirus-encoded exonuclease (ExoN) activity residing in nsp14. Interestingly, like the EndoU, the coronavirus ExoN is an integral component of the coronaviral RTC, and ExoN has been demonstrated to provide an RNA proofreading function that permits coronaviruses to stably maintain their extraordinary large RNA genome [53–55]. A functional link between the two coronaviral ribonucleases, ExoN and EndoU, would suggest an unprecedented concept of viral RNA quality/decay control that goes beyond RNA proofreading and includes the removal of dsRNA-based PAMPs at the site of RNA synthesis to efficiently evade innate and intrinsic antiviral host cell responses.

## Materials and methods

### Viruses

Recombinant MHV strain A59, HCoV-229E, MHV_H277A_, HCoV-229E_H250A_, and MHV_D130A_ were generated using the vaccinia virus-based reverse genetic system as previously described [9, 56–58]. Viruses were propagated on 17Cl1 mouse fibroblasts (MHV) and on Huh-7 hepacarcinoma cells (HCoV-229E) and their identity was confirmed by sequencing.

### Mice and viral infection

C57BL/6 mice were purchased from Charles River, from Jackson Laboratories and from the Department of Clinical Research, Bern, Switzerland (BE103/15). All genetically modified mice were produced on an inbred C57BL/6 background. Mice were bred and maintained at the Cantonal Hospital in St. Gallen (Mda5^-/-^, TLR7^-/-^, Mda5^-/-^ / TLR7^-/-^, IFNAR^-/-^); at the Heimholtz Centre for Infection Research, Braunschweig or at the Twincore, Centre for Experimental and Infection Research, Hannover (MAVS^-/-^, IRF3/5/7^-/-^); Cleveland Clinic Lerner Research Institute, Cleveland (RNase L^-/-^, [59]); at the Friedrich-Loeffler Institut, Greifswald (PKR^-/-^); at the Department of Cancer Biology, Lerner Research Institute, Cleveland (RNase L ^-/-^ / PKR^-/-^, [34]). Mice were maintained in single ventilated cages in groups of maximally 5 individuals, were fed ad libitum and observed daily. All animal experiments were carried out in accordance with the animal legislation of the respective countries and their approval from the animal studies committees.

To monitor viral spread *in vivo*, mice at the age of 8–10 weeks were injected intraperitoneally with 500 plaque-forming units (pfu) of MHV-A59 and MHV_H277A_, diluted in MEM2% (2% heat-inactivated fetal calf serum, penicillin (60 μg/ml) and streptomycin (100 μg/ml)). Mice were euthanized two days post infection (d.p.i.) and liver and spleen were harvested, weighed and homogenized. Viral load (pfu/g organ) was determined by plaque assay.

### Cells

Murine L929 fibroblasts (Sigma), 17Cl1 cells (gift from S.G. Sawicki) were cultured in MEM10%. Murine embryonic fibroblasts (C57BL/6 MEFs) were maintained at low passage in DMEM10% (Dulbecco’s Modified Eagle Medium-GlutaMAX). Huh-7 cells (gift from V. Lohnmann) were cultured in DMEM5% and 0.5mM sodium pyruvate. MRC-5 cells (human lung fibroblast-like cells; Sigma) were maintained at low passage in MEM10%, supplemented with 1% non-essential amino acids (NEAA). HEK293-Mx1-Luc cells (gift from G. Kochs) were maintained in DMEM10%, supplemented with G418 (200 μl/ml)[60].

### Isolation of murine and human primary macrophages

Murine bone marrow-derived macrophages were obtained from mice at the age of 8–12 weeks. Progenitor cells were isolated from hind limbs, passed through a cell strainer and red blood cell lysis was carried out in 1 ml lysis buffer/mouse (0.15 M NH_4_Cl, 1 mM KHCO_3_, 0.1 mM EDTA). Cells were washed 3x with PBS and taken up in macrophage medium (IMDM Iscove’s Modified Dulbecco’s Medium, 5–10% M-CSF (L929-supernatant), 0.1% 50 mM 2-mercaptoethanol). New medium was added 3 d.p.i. and adherent cells were harvested 7 d.p.i. Primary human macrophages were obtained from peripheral blood of healthy human donors as previously described[61]. Peripheral blood mononuclear cells were isolated by centrifugation of buffy coat blood over a Leucosep tube (Greiner Bio One). Cells from the enriched interphase were collected, washed twice with PBS and red blood cells were removed. Cells were taken up in IMDM and plated in 24-well cell culture plates. Non-adherent cells were removed three h.p. seeding and adherent cells were cultured for 14 days in IMDM30%. Medium was changed every second day. All experiments using human blood were in accordance with the Swiss federal legislation and the institutional guidelines of the Cantonal Hospital St. Gallen and the Blutspendedienst SRK Bern.

### Viral infections and measurement of viral burden

Murine cells were infected with MHV-A59 and MHV_H277A_ (MOI = 0.1 L929 cells; or 1 MEFs, L929 cells, macrophages) at 37°C. Virus inoculum was removed 2 h.p.i., cells were washed with PBS and fresh medium was added. Virus supernatant was harvested and cellular RNA was collected in TRIzol (Invitrogen). Human macrophages (counted at the day of infection) and MRC5 cells were infected with HCoV-229E and HCoV-229E_H250A_ (MOI = 1), virus inoculum was removed 2 h.p.i. (macrophages) and 4 h.p.i (MRC5), cells were washed and supernatant was harvested 24 h.p.i (macropahges) and 48 h.p.i (MRC5). Viral titer in the supernatant was determined by standard plaque titration on L929 cells (MHV) and Huh7 cells (HCoV-229E).

### RNA isolation and quantitative RT-PCR

Total cellular RNA was isolated from murine macrophages with TRIzol (Life Technologies) and genomic DNA was removed with DNase (Ambion, DNA-free DNase Treatment). RNA concentration was measured by nanodrop and input for cDNA synthesis was standardized to 300 ng. Synthesis of cDNA was carried out using the M-MLV reverse transcriptase from Promega and the 20 μl cDNA were diluted with 80 μl dH_2_O. The FastStart Universal SYBR Green Master (Rox) Mix (Roche) was used for measuring mRNA expression of IFN-β, GAPDH and Tbp (S1 Table). Induction of IFN-β was normalized to levels of the household genes GAPDH and Tbp (geometric mean) and expressed as ΔΔC_T_ over mock (ΔC_T_ values calculated as C_T_ reference—C_T_ target) [62]. Expression of OAS1a, OAS2, OAS3 and RNase L mRNA in mock infected macrophages and L929 cells was normalized to levels of GAPDH [63] (S1 Table). Expression levels were displayed as ΔC_T_ values (C_T_ reference—C_T_ target). Copy number of cell-associated viral RNA isolated from MHV infected macrophages was determined using the RT TaqMan PCR system (TaqMan Fast Universal PCR Master Mix (2x), No AmpErase UNG, Applied Biosystems) with primers and probe specific to the MHV genome fragment encoding the nucleocapsid (S1 Table). Copy numbers were determined by using a standard curve, consisting of an *in vitro* transcribed RNA of known copy number, obtained from a plasmid comprising the MHV-nucleoprotein sequence.

### Quantitative RT-PCR to determine MHV genomic RNA and subgenomic mRNA

Two RNA standards encompassing MHV nucleotides (nts) 15–530 (genomic standard), and MHV nts 15-63/21746-22259 (corresponding to the MHV mRNA2 leader-body junction; subgenomic mRNA2 standard) were prepared as follows. One RT-PCR product corresponding to the 5’ region of MHV-A59 genomic RNA was generated by RT-PCR using viral RNA from MHV-A59 infected cells as template and primers T7-MHV-leader15-frw and MHV-ORF1-rev530 (S2 Table). The resulting RT-PCR product comprised the T7-RNA-Polymerase promoter, MHV nts 15–530. A second RT-PCR product corresponding to the 5’ region of MHV-A59 mRNA2 was generated by RT-PCR using viral RNA from MHV-A59 infected cells as template and primers T7-MHV-leader15-frw and MHV-ns2-rev22259 (S2 Table). The resulting RT-PCR product comprised the T7-RNA-Polymerase promoter, MHV nts 15–63, and MHV nts 21746–22259. Both RT-PCR products were separated on an agarose gel and DNA fragments of the appropriate size were excised and purified. *In vitro* transcribed (IVT) RNA was prepared using the RiboMAX Large Scale RNA Production System–T7 (Promega). RQ1 RNase-free DNase was added to the IVT RNA and incubated for 15 minutes at 37°C. The *in vitro* transcribed RNA was purified using the NucleoSpin RNA kit (Macherey-Nagel), its quantity was determined (absorbance at 260nm) and eight 10-fold dilutions were prepared. Synthesis of cDNA was carried out for each dilution using the M-MLV reverse transcriptase and random primers (Promega). The 20 ul of cDNA were diluted with 80 μl dH_2_O and used as a standard for the quantitative RT-PCR reaction.

Copy numbers of genomic and subgenomic viral RNA were determined for samples obtained from C57BL/6 macrophages infected with MHV-A59 and and MHV_H277A_ (MOI = 1). A multiplex reaction (TaqMan Fast Universal PCR Master Mix (2x), No AmpErase UNG, Applied Biosystems) and primers and a probe specific to the genomic or subgenomic sequence (S2 Table), respectively, were used.

### IFN-β Enzyme-Linked Immunosorbent Assay (ELISA), IFN-bioassay and IFN-pre-treatment

Total type-I IFN in supernatants obtained from MHV infected macrophages was measured by an IFN-β enzyme-linked immunosorbent assay (BPL Assay Science, VeriKine Mouse IFN Beta ELISA Kit, 15.6–1000 pg/ml). Technical replicates were pooled. The level of biologically active human type I IFN in the supernatant of infected human macrophages was measured with HEK293 cells that were stably transfected with a luciferase reporter plasmid under the control of the Mx-promoter [60, 64]. Recombinant IFNα A/D (Sigma) was used as a cytokine standard and luciferase activity was detected 16 hours p.i. by a Luminometer (Luciferase Assay System, Promega). To assess the sensitivity of MHV and HCoV-229E towards IFN-I, L929 cells (MHV) and MRC5 cells (HCoV-229E) were treated with recombinant IFNα A/D (Sigma, as indicated in the figures) for four hours, and then infected with MHV-A59, MHV_H277A_, MHV_D130A_, HCoV-229E and HCoV_H250A_ (MOI = 1). Virus supernatant was harvested 24 h.p.i (MHV) and 48 h.p.i (HCoV-229E). Viral replication was measured by plaque assay (MHV) or by using primers and a probe specific to the HCoV-229E membrane protein (S1 Table) and the QuantiTect Probe OneStep RT-PCR Kit (Qiagen). To assess baseline expression, L929 cells were pre-treated with 12.5 U of IFNα A/D for 16h and then infected with MHV-A59 and MHV_H277A_ (MOI = 1). Cellular RNA was isolated with TRIzol, cDNA was prepared and qRT-PCR was performed (S1 Table).

### Measurement of RNA quality with Fragment Analyzer

Total RNA isolated from MHV-infected macrophages and L929 cells was analysed with a Fragment Analyzer (Labgene) using the DNF-471 standard sensitivity RNA analysis kit (15nt lower marker, Advanced Analytical Technologies).

### Flow Cytometry (FACS)

To assess the amount of dsRNA-positive cells, C57BL/6 and IFNAR^-/-^ macrophages (5x10^6^ cells) were infected with MHV-A59 and MHV_H277A_ (MOI = 1). For FACS, cells were detached with PBS at 4°C, centrifuged and fixed with 4% formalin. Cells were permeabilized with 0.1% Triton and stained with the mouse monoclonal antibody J2 directed against dsRNA (1:200, English & Scientific Consulting Bt) and the anti-MHV nsp2/3 rabbit antiserum[65] (1:600) at 4°C for 1h. Cells were washed, stained with a secondary antibody Goat F(ab’)2 anti-mouse IgG2a, human ads-PE (1:200, SouthernBiotech) and a donkey anti-rabbit Alexa-647 (1:400) for 30min. FACS was performed using the BD FACS Canto II and data were analysed using FlowJo v.10. Cell debris was excluded based on a gate of FSCA/SSCA, followed by a doublet discrimination FSCA and FSW. To assess the extent of translation inhibition, C57BL/6 macrophages were infected with MHV-A59 and MHV_H277A_ (MOI = 1). 15 min prior to each time point, puromycin (Sigma) was added to the wells at a final concentration of 20μg/ml to label active translation. At the indicated time points, cells were washed twice with PBS, and subsequently detached with PBS at 4C. Cells were fixed in 2% PFA for 30min at RT, and washed once with FACS buffer (PBS + 1% BSA). Cells were incubated in ice-cold methanol for 10min at 4C. After two wash steps in FACS buffer, cells were incubated with a primary mouse antibody directed against puromycin (1:100, Milipore) and a primary rabbit anti-eIF2α-P (Abcam; 1:100) in FACS buffer for 45min at RT. Cells were washed twice with FACS buffer and incubated with the secondary antibody donkey anti-mouse-Alexa488 (1:200), and donkey anti-rabbit-Alexa648 (1:200) in FACS buffer for 45min at RT in the dark. Cells were washed once in FACS buffer, and kept in 1% PFA in the dark until cells were analysed with the FACS Canto (BD) using the BD FACS Diva software.

### Statistical analysis of data

Kinetics of virus growth, viral RNA and IFN-β mRNA were analyzed using non-linear regression. The regression model is an exponential saturation model (increasing response with constant asymptote) that additionally allows a peak response. It is described by the formula

$$Y\left(T\right)=A_{G}-e^{\frac{M_{G}-T}{S}}+P_{G}\cdot e^{-{\left(\frac{M_{G}-T}{S}\right)}^{2}}$$

where *Y* is the response value (log virus titer or log expression value), *T* is the time (in hours), *A* is the value of the asymptote, *M* is a „midpoint value”representing the time where the exponential increase has reached half of the asymptotic value and where the peak is located, *P* is a value describing the additional peak height, and *S* is a scale parameter specifying the steepness of the exponential increase and the width of the peak. The coefficients *A*, *M* and *P* were determined individually for the groups to be compared (MHV-A59 and MHV_H277A_), as symbolized by the index *G*. The model was chosen on pragmatic grounds because it was able to describe the time courses of all data very well and the coefficients represent biologically relevant aspects of the kinetics that can be addressed directly by statistical tests. The analyses were performed in R, version 3.2.3 [66] with the function nls [67] using the algorithm “Port” restricting the coefficients to positive values. P-values and confidence intervals were determined by parametric bootstrapping, resampling the residuals from a normal distribution with mean 0 and variance estimated from the variance of residuals of the fitted model. Confidence bands were generated by connecting the point-wise 95% confidence intervals of the predictions. The significance of the difference between treatments in differences between maxima of the groups (i.e., the Group-Treatment interaction) was determined by bootstapping the difference-in-difference. The assumption of normally distributed residuals was checked and confirmed with normal-quantile quantile plots.

All other data were analysed using R v. 3.0.3 (R Development Core team, 2008) and displayed using GraphPad Prism v.5 and CorelDraw Graphic Suite X4. The types of statistical tests used are indicated in the corresponding figure legends.

### Ethics statement

C57BL/6 mice, Mda5^−/−^ mice, TLR7^−/−^ mice, Mda5^−/−^/TLR7^−/−^ mice, IFNAR^−/−^ mice, were bred and maintained in the Kantonsspital St.Gallen animal facility and the University of Bern animal facility in accordance with federal and cantonal guidelines (Tierschutzgesetz). The protocols were approved by the Kantonal Veterinary Offices of the Kanton St.Gallen (SG09/87 and SG09/14 and SG10/14) and Kanton Bern (BE103/15).

C57BL/6 mice, and RNase L^−/−^, and RNase L^−/−^/PKR^−/−^ mice were bred and maintained in the University of Pennsylvania and the Cleveland Clinic animal facilities. Protocols were in accordance with the National Institutes of Health (United States) Public Health Service (PHS) Policy on Humane Care and Use of Laboratory Animals, revised 2015, and approved by the Federal Government Animal Welfare Assurance for the University of Pennsylvania (Reference Assurance # A3079-01) and Institutional Animal Care and Use Committee at the University of Pennsylvania and the Federal Government Animal Welfare Assurance for *the* Cleveland Clinic (Reference Assurance # A3145-01).

C57BL/6 mice, MAVS^-/-^, and mice, IRF3/5/7^-/-^ mice were bred and maintained in the animal facility of the TWINCORE, Hannover, Germany, and the protocols were approved by the Niedersächsisches Landesamt für Verbraucherschutz und Lebensmittelsicherheit.

PKR^−/−^ mice were bred and maintained in the animal facility of the Friedrich-Loeffler-Institute, Insel Riems, and the protocols were approved by the Landesamt für Landwirtschaft, Lebensmittelsicherheit und Fischerei (LALLF) Mecklenburg-Vorpommern (LALLF 7221.3–2.1-011/13.).

All experiments using human blood (buffy coat) were in accordance with the Swiss federal legislation and the institutional guidelines (including informed consent) of the Cantonal Hospital St. Gallen and the Blutspendedienst SRK Bern.

## Supporting information

S1 Fig The sensitivity of MHV_H277A_ towards IFN-I is in the range observed for the highly sensitive 2’O-methyltransferase mutant. Comparative analysis of the sensitivity of MHV-A59, MHV_H277A_ and MHV_D130A_ (lacking the 2’-O methyltransferase activity) to IFN-I pre-treatment. L929 cells were pre-treated with various dosages of type-I IFN for four hours. Thereafter, cells were infected at an MOI of 1 and virus titers in the supernatant were measured at 24 h.p.i. by plaque assay. Data represent two independent experiments. Data from MHV-A59 and MHV_H277A_ were already displayed in Fig 4C. Data are displayed as differences to untreated controls. Mean and SEM are displayed.
(TIF)

S2 Fig EndoU deficient mutant triggers rRNA breakdown in MAVS^-/-^ and IRF3/5/7^-/-^ macrophages. **(a)** Degradation of rRNA in MHV-A59 and MHV_H277A_ infected (MOI = 1) MAVS^-/-^ and IRF3/5/7^-/-^ bone marrow-derived macrophages. Total RNA was isolated at indicated time points and degradation of ribosomal RNA was assessed with a Fragment Analyzer. One representative picture and migration of 18S and 28S ribosomal RNA is displayed. The RNA Quality Number (RQN) is indicated. **(b)** Cell-associated viral RNA following infection of C57BL/6 bone marrow-derived macrophages (MOI = 1) with MHV-A59 and MHV_H277A_. Copy numbers of genomic RNA (gRNA) and subgenomic RNA (mRNA2) are indicated. Data represent five independent experiments with mean and SEM displayed.
(TIF)

S3 Fig Assessment of OAS1a, OAS2, OAS3 and RNase L mRNA baseline expression. Baseline expression levels of OAS1a, OAS2, OAS3, RNase L mRNA in C57BL/6, Mda5^-/-^, MAVS^-/-^, RNase L^-/-^, PKR^-/-^, IRF3/5/7^-/-^, IFNAR^-/-^, and RNase L^-/-^/PKR^-/-^macrophages was assessed by qRT-PCR. Levels of mRNA expression relative to GAPDH are shown as log_10_(2^ΔCT^), where ΔC_T_ = (C_T_ reference—C_T_ target). Bars in black highlight macrophage genotypes that restrict the replication of MHV_H277A_, while white bars highlight macrophage genotypes that at least partially permit MHV_H277A_ replication. Statistical comparisons were carried out between mRNA expression levels measured in C57BL/6 cells versus knockout cells. Mean and SEM are displayed. Data points that show significance differences in a two-sided, unpaired Student’s t-test are depicted: * p < 0.05, ** p < 0.01, *** < 0.001. ND, not detected.
(TIF)

S4 Fig EndoU-deficient MHV induces elevated expression of IFN-β and assessment of OAS1a-3 and RNase L mRNA expression levels in L929 cells. (**a**) Replication kinetics of MHV-A59 and MHV_H277A_ following infection (MOI = 1) of L929 cells, which were left untreated (left panel) or were pre-treated for 16h with 12.5 U of IFN-I (right panel). Data represent four independent experiments, each performed in duplicates. Mean and SEM are depicted. The 95% confidence bands are highlighted in grey. Statistically significant comparisons are displayed; *** p < 0.001. The differences in peak levels of IFN-β expression (left panel: MHV-A59: 6.2, MHV_H277A_: 13.6; right panel: MHV-A59: 7.9, MHV_H277A_: 15.9) were statistically significant (***; p = <0.001). (**b**) Analysis of baseline expression levels of OAS1a, OAS2, OAS3, and RNase L mRNA by qRT-PCR of L929 cells that were left untreated (black bars) or were pre-treated with 12.5 U of IFN-I for 16h (white bars). Levels of mRNA expression relative to GAPDH are shown as log_10_(2^ΔCT^), where ΔC_T_ = (C_T_ reference—C_T_ target). Data represent four independent experiments, each performed in duplicates. Mean and SEM are displayed. Data points that show significant differences in a two-sided unpaired Student’s t-test are depicted: * p < 0.05, ** < 0.01.
(TIF)

S1 Table Primers and probes used in qRT-PCR. (PDF)

S2 Table **(A)** Preparation of genomic and subgenomic RNA standards. **(B)** Primers and probes used in the multiplex qRT-PCR reaction.
(PDF)
