# Supplementary material for: Early endonuclease-mediated evasion of RNA sensing ensures efficient coronavirus replication
Source: PLoS Pathog. 2017 Feb 3;13(2):e1006195. doi: 10.1371/journal.ppat.1006195 (PMC5310923; doi:10.1371/journal.ppat.1006195)
Supplement: S1 Table — (PDF) [file ppat.1006195.s005.pdf]

Supplemental Table 1 Primers and probes used in qRT-PCR.

Gene	forward 5'-3'	reverse 5'-3'	probe 5-'3'
IFNβ	AGAAAGGACGAACATTCGGAAA	TCCGTCATCTCCATAGGGATCTT	
GAPDH	TGCACCACCAACTGCTTAG	GGATGCAGGGATGATGTTC	
TATA-box binding protein (Tbp)	ACGGACAACTGCGTTGATTTT	ACTTAGCTGGGAAGCCCAACTT	
OAS1a	GGGAACAAGGGAATGGATGTTATGAGT	ATTTGGAGACCTCCTGGTGTTGAAAGT	
OAS2	CTGGCTGACTGGAAACTGGTCATCT	TTCAGACAGTTTTCGTTGGGTGTGA	
OAS3	CACTGTTCTGGAGCTGATTGTCCAGT	GGTCTTGTCTTCTGCGCTGTAGTTGA	
RNase L	AGCCCAATCCCTACTCCAAGACTCTC	ATCTTCGACAACGGTCCTCTGACTTC	
nucleocapsid MHV	GCCTCGCCAAAAGAGGACT	GGGCCTCTCTTTCCAAAACAC	FAM- CAAACAAGCAGTGCCCAGTGCAGC-BHQ1
membrane protein HCoV-229E	TTCCGACGTGCTCGAACT	CCAACACGGTTGTGACAG	FAM-TGGGCATGGAATCCTGAG-BHQ1
