# Supplementary material for: Early endonuclease-mediated evasion of RNA sensing ensures efficient coronavirus replication
Source: PLoS Pathog. 2017 Feb 3;13(2):e1006195. doi: 10.1371/journal.ppat.1006195 (PMC5310923; doi:10.1371/journal.ppat.1006195)
Supplement: S2 Table — (A) Preparation of genomic and subgenomic RNA standards. (B) Primers and probes used in the multiplex qRT-PCR reaction. (PDF) [file ppat.1006195.s006.pdf]

Supplemental Table 2A Preparation of genomic and subgenomic RNA standards

standard	name	start 5' in MHV-A59*	end 3' in MHV-A59*	primer sequence 5'-3'
genomic and subgenomic	T7-MHV-leader15-frw	15	53	ACTG**TAATACGACTCACTATAG**GGCGTCCGTACGTACCCTCTCAACTCTAAAACTCTTGTA
genomic	MHV-ORF1-rev530	530	509	GGCTTAACCAAGACGGCACTAC
subgenomic	MHV-ns2-rev22259	22259	22241	TCAAGCCCAGCATCCGTTA

* AY700211
T7 sequence is indicated in bold

Supplemental Table 2B Primers and probes used in the multiplex qRT-PCR reaction

decription	name	start 5' in MHV-A59*	dye	primer sequence 5'-3'	end 3' in MHV-A59*	quencher
Frw Primer Genomic and Subgenomic RNA	Primer MHV-A59-L25frw	25		CGTACCCTCTCAACTCTAAAACTCTTGTA	53	
Rev Primer Genomic RNA	Primer MHV-A59-5utr143rev	164		GGCAGAGAACGAAAGTCAAGGA	143	
Probe Genomic RNA	Probe MHV-5UTR-103	103	Yakima Yellow	CCCGCGGGCCTGGTCTTGTC	122	BHQ-1
Rev Primer Subgenomic RNA	Primer MHV-ns2-21778rev	21801		GAAATGATTAGGCTTGTCAGCAAA	21778	
Probe Subgenomic RNA **	Probe MHV-ns2-sg-MGB	60-69&21752-21765	FAM	ATCTAATCTATACTTGTCGTGGCT	**	MGB

* AY700211
** overlapping Sequence Leader/ns2 subgenomic
